# H4K20me1 Contributes to Downregulation of X-Linked Genes for *C. elegans* Dosage Compensation

**DOI:** 10.1371/journal.pgen.1002933

**Published:** 2012-09-13

**Authors:** Anne Vielle, Jackie Lang, Yan Dong, Sevinc Ercan, Chitra Kotwaliwale, Andreas Rechtsteiner, Alex Appert, Q. Brent Chen, Andrea Dose, Thea Egelhofer, Hiroshi Kimura, Przemyslaw Stempor, Abby Dernburg, Jason D. Lieb, Susan Strome, Julie Ahringer

**Affiliations:** 1 The Gurdon Institute and Department of Genetics, University of Cambridge, Cambridge, United Kingdom; 2 Department of Molecular, Cell, and Developmental Biology, University of California Santa Cruz, Santa Cruz, California, United States of America; 3 Department of Biology, Carolina Center for the Genome Sciences, Lineberger Comprehensive Cancer Center, The University of North Carolina at Chapel Hill, Chapel Hill, North Carolina, United States of America; 4 Center for Genomics and Systems Biology, New York University, New York, New York, United States of America; 5 Department of Molecular and Cell Biology and California Institute for Quantitative Biosciences (QB3), University of California Berkeley, Berkeley, California, United States of America; 6 Howard Hughes Medical Institute, Chevy Chase, Maryland, United States of America; 7 Graduate School for Frontier Biosciences, Osaka University, Osaka, Japan; Emory University, United States of America

## Abstract

The *Caenorhabditis elegans* dosage compensation complex (DCC) equalizes X-chromosome gene dosage between XO males and XX hermaphrodites by two-fold repression of X-linked gene expression in hermaphrodites. The DCC localizes to the X chromosomes in hermaphrodites but not in males, and some subunits form a complex homologous to condensin. The mechanism by which the DCC downregulates gene expression remains unclear. Here we show that the DCC controls the methylation state of lysine 20 of histone H4, leading to higher H4K20me1 and lower H4K20me3 levels on the X chromosomes of XX hermaphrodites relative to autosomes. We identify the PR-SET7 ortholog SET-1 and the Suv4-20 ortholog SET-4 as the major histone methyltransferases for monomethylation and di/trimethylation of H4K20, respectively, and provide evidence that X-chromosome enrichment of H4K20me1 involves inhibition of SET-4 activity on the X. RNAi knockdown of *set-1* results in synthetic lethality with dosage compensation mutants and upregulation of X-linked gene expression, supporting a model whereby H4K20me1 functions with the condensin-like *C. elegans* DCC to repress transcription of X-linked genes. H4K20me1 is important for mitotic chromosome condensation in mammals, suggesting that increased H4K20me1 on the X may restrict access of the transcription machinery to X-linked genes via chromatin compaction.

## Author Summary

In many animals, males have one X chromosome and females have two. However, the same amount of gene expression from X chromosomes is needed in the two sexes. The process of dosage compensation (DC) globally regulates X-chromosome gene expression to make it equal between the sexes, and it occurs in different ways in different animals. In mammals, one X chromosome in females is randomly inactivated, leaving one active X chromosome. In contrast, in the nematode worm *C. elegans*, the two X chromosomes in hermaphrodites are repressed two-fold to match gene expression to the single X chromosome in males. Previous work in *C. elegans* identified proteins required for DC that bind to the X chromosome, but their mode of action is not known. Here we show that DC proteins lead to higher levels of histone H4 lysine 20 monomethylation (H4K20me1) on hermaphrodite X chromosomes and that H4K20me1 functions in repressing X-chromosome gene expression. This shows that histone modification is an important aspect of the mechanism of dosage compensation. Together with previous work linking H4K20me1 to chromatin structure regulation, our results suggest that dosage compensation might lower gene expression on hermaphrodite X chromosomes by compacting them.

## Introduction

In many animals, males and females have a different number of X chromosomes. Dosage compensation is a chromosome-wide process of gene regulation that equalizes gene expression between the sexes despite the difference in X-linked gene dosage, and is achieved by a variety of mechanisms [1], [2]. In humans, one X chromosome is inactivated in females. In *Drosophila*, expression from the single X chromosome in males is upregulated two-fold to match expression from the two X chromosomes in females. In *C. elegans*, the two X chromosomes in hermaphrodites are downregulated two-fold to match expression from the single X chromosome in males. In each of these cases, regulation of gene expression involves the targeting of specialized protein complexes specifically to the X chromosome. Studies of different dosage compensation mechanisms have uncovered chromatin-mediated mechanisms of gene regulation.

In *C. elegans*, dosage compensation is achieved by the dosage compensation complex (DCC) (reviewed in [3]). The core of the DCC consists of a five-subunit condensin complex named condensin I^DC^. Condensin complexes mediate chromosome condensation and resolution in mitosis and meiosis, and participate in crossover control during meiosis [4]. Four subunits (MIX-1, DPY-26, DPY-28, and CAPG-1) are shared with canonical condensin I, while DPY-27 is specific to condensin I^DC^ [5]. The central role of a condensin-like complex in dosage compensation suggests that the mechanism of dosage compensation involves regulation of chromatin structure. Five other components of the DCC (SDC-1, SDC-2, SDC-3, DPY-21 and DPY-30) physically interact with different subunits of condensin I^DC^, and all examined components of the DCC are enriched on hermaphrodite X chromosomes relative to autosomes [3].

The DCC is targeted to the X chromosome through specific sequence elements, called *rex* (recruitment elements on X) sites (reviewed in [3]). After recruitment, the DCC spreads to *dox* (dependent on X) sites, which consist mostly of active promoters. The zinc finger protein SDC-2 is the primary X-chromosome recruitment factor for the DCC. The DCC also binds to some autosomal sites at lower levels, but the functional significance of autosomal binding is not yet known [6], [7]. SDC-3 requires SDC-2 for X-chromosome binding, and all of the other DCC components require SDC-2 and SDC-3 for recruitment. Loss of DCC proteins impairs dosage compensation, resulting in upregulation of X-linked genes and death of XX animals. DPY-21 and SDC-1 null mutants have milder dosage compensation defects and are viable, with apparently normal DCC protein localization on X [8], [9], [10], [11], [12].

A current model is that SDC proteins recruit condensin I^DC^ to the X chromosome, leading to changes in chromatin structure that result in reduction of gene expression. In XX animals, not all genes on X are dosage compensated, and DCC association with genes correlates with gene expression level but not with the degree of repression [7]. The mechanism of repression is not understood, but DCC mutants have increased levels of RNA polymerase II on X-linked genes, indicating regulation at the level of transcription [13].

Here we show that the DCC is required for enrichment of the histone modification H4K20me1 on the X chromosomes of hermaphrodites and that the responsible histone methyltransferase SET-1 is important for downregulation of dosage-compensated genes. Our study implicates the histone modification H4K20me1 in the process of dosage compensation.

## Results

**Figure 1:**
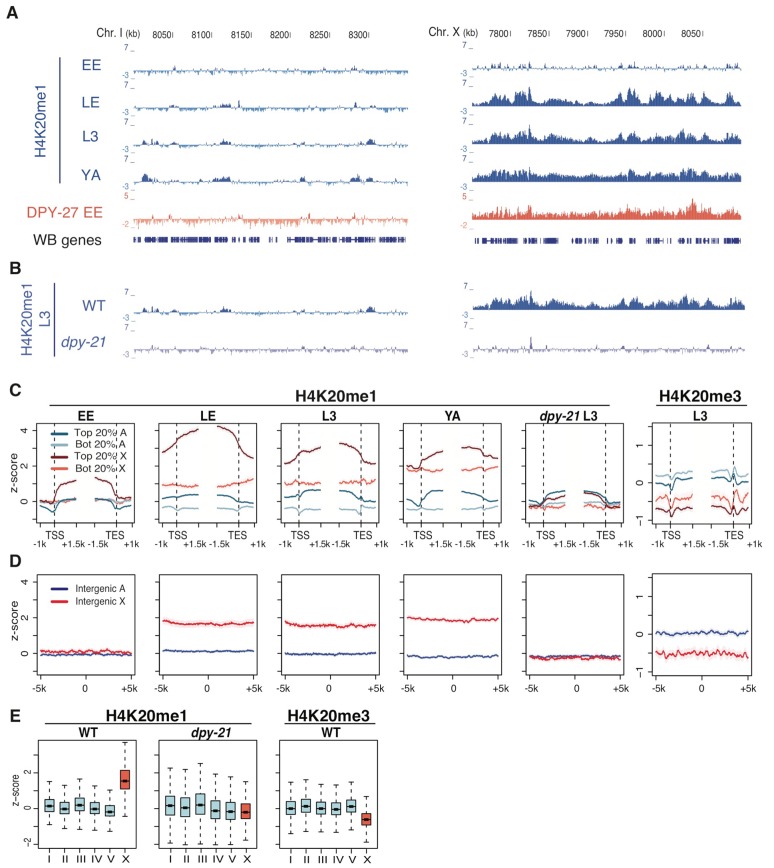
H4K20me1 becomes enriched on the X chromosome in wild type but not in a mutant defective in dosage compensation. (A) Genome browser tracks of indicated ChIP signals across representative regions of chromosome I and chromosome X in wild-type early embryos (EE), late embryos (LE), L3 larvae (L3), and *fem-2* female young adults (YA). For each track, the average of two independent biological replicates is shown as z-scores (standardized log_2_ ratios of ChIP/Input signals). High H4K20me1 enrichment on X begins in late embryos. A track of DPY-27 (a DCC component) is shown for reference. (B) Enrichment of H4K20me1 on the X is lost in *dpy-21* mutants. (C) Plots of H4K20me1 and H4K20me3 signal across the TSS (transcript start site) and TES (transcript end site) of genes on X (red) and autosomes (blue). Genes in the top 20% of expression (dark shades) and bottom 20% (light shades) are plotted separately. (D) Plots of H4K20me1 and H4K20me3 signal centered at intergenic regions at least 5 kb from any annotated feature. (E) Box plots of overall H4K20me1 and H4K20me3 signals on each chromosome. Each box shows the median and extends from the 25^th^ to the 75^th^ percentile of the z-scores in the set; whiskers extending from the box indicate the 2.5^th^ and 97.5^th^ percentiles.

The localization of dosage compensation proteins to the X chromosome become apparent at around the 30-cell stage of embryogenesis [3], [14]. Our previous experiments mapping the genome-wide patterns of H4K20me1 suggested that this histone modification might function in dosage compensation: H4K20me1 is weakly enriched on the X chromosome in early embryo populations that span initiation of dosage compensation (1–300-cell stages with 76%<100-cell) and strong enrichment at the third larval (L3) stage, when dosage compensation is fully active (Figure 1A) [15]. We performed additional ChIP experiments to better define the timing of H4K20me1 enrichment on X. We found that late embryos (99% 100-cell to 3-fold stage), which have activated dosage compensation, display high H4K20me1 on the X, similar to the pattern in L3 larvae (Figure 1A). Moreover, enrichment on the X chromosome is maintained in adult hermaphrodites (Figure 1A). Therefore, H4K20me1 is enriched on the X chromosome at developmental stages when dosage compensation is active.

### H4K20me1 is enriched in both coding and non-coding regions along the X chromosome in dosage-compensating XX hermaphrodites

In *C. elegans*, dosage compensation downregulates genes on the X chromosome. We therefore examined the pattern of H4K20me1 with respect to gene features. As seen previously in early embryos and L3 larvae [15], H4K20me1 is enriched across active gene regions at all developmental stages examined, with high enrichment on X-linked genes and lower enrichment on autosomal genes (Figure 1C). H4K20me1 levels are positively correlated with transcript levels for both X-linked and autosomal genes (r_s_ = 0.66 and r_s_ = 0.56, respectively). Notably, inactive X-linked genes have higher levels of H4K20me1 than active autosomal genes (Figure 1C). On autosomes H4K20me1 is primarily confined to transcribed regions, while on the X, H4K20me1 is elevated across the chromosome, including regions that are more than 5 kb from any annotated gene feature (Figure 1D). This widespread distribution of H4K20me1 on the X chromosome suggests a role in chromosome-wide regulation of gene expression.

### H4K20me1 is enriched on the X after the onset of dosage compensation

**Figure 2:**
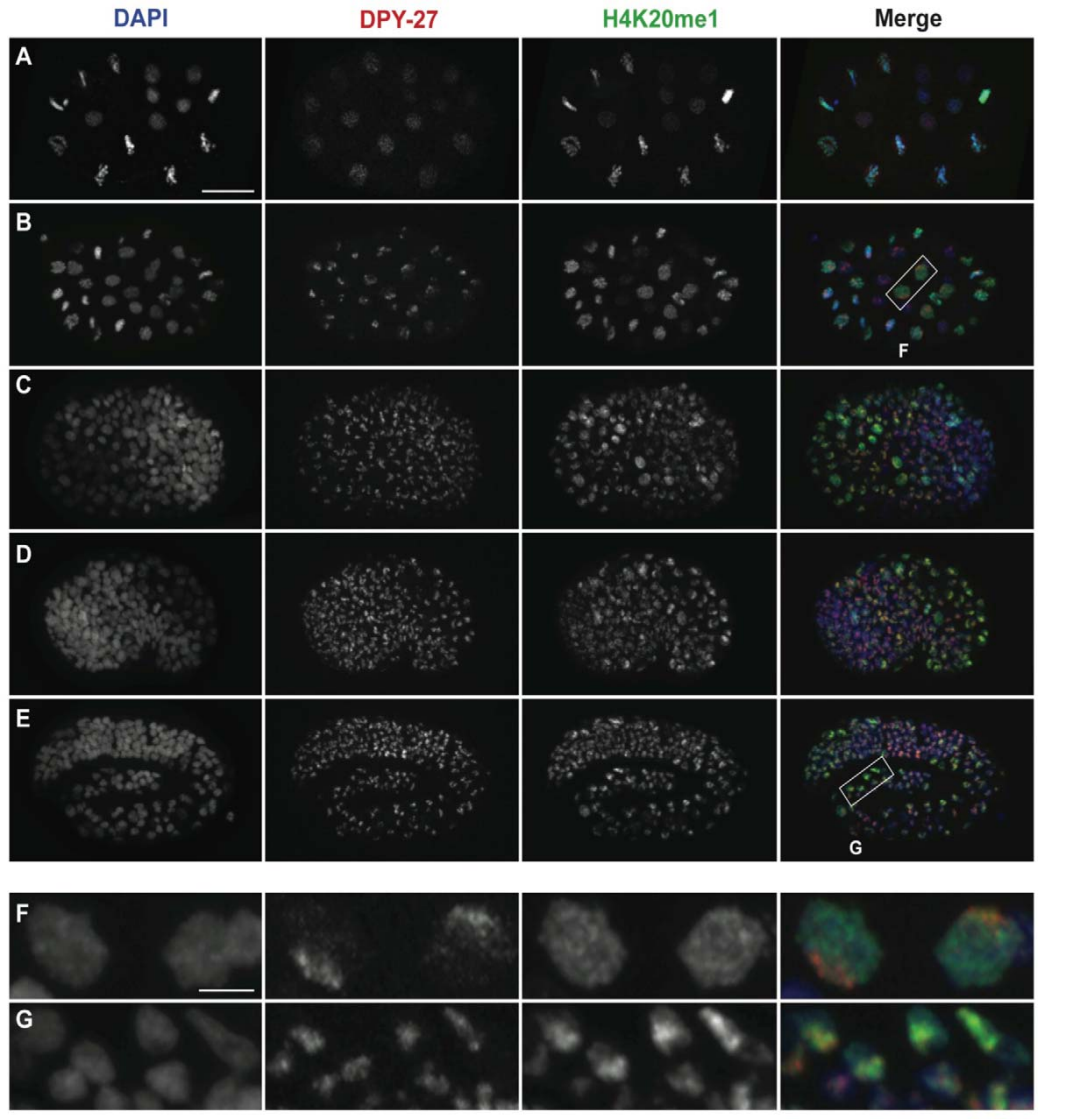
H4K20me1 becomes concentrated on the X chromosome after the DCC. Wild-type embryos at (A) 20-cell, (B) 40-cell, (C) bean, (D) comma, and (E) three-fold stages stained for DNA (DAPI, blue, left column), DPY-27 (red, second column), and H4K20me1 (green, third column). Boxed regions in B and E are enlarged in F and G, respectively. In very early embryos (A) DPY-27 exhibits weak and diffuse nuclear staining while H4K20me1 is diffuse in interphase nuclei and associated with condensed chromatin during mitosis. By the ∼40-cell stage (B, F) DPY-27 is enriched on the X chromosome, while H4K20me1 continues to show a uniform nuclear distribution. Comma-stage embryos (D) contain some nuclei with H4K20me1 enrichment on the X. This enrichment becomes more apparent as embryogenesis progresses, and by the 3-fold stage (E, G) H4K20me1 is concentrated in bright foci that co-localize with DPY-27 on the X. Scale bars represent 10 um (A–E) and 2 um (F, G). Monoclonal antibody15F11 was used to detect H4K20me1.

Our ChIP experiments from early and late stage embryos, L3s, and young adults showed that the timing of H4K20me1 enrichment on the X chromosome is consistent with a role in dosage compensation. To determine more precisely when H4K20me1 becomes enriched on the X chromosome, we performed immunofluorescence experiments using two different antibodies specific for H4K20me1. In embryos prior to the 30-cell stage, neither DPY-27 nor H4K20me1 was concentrated in any subnuclear region (Figure 2A). However, we observed that nuclear staining of H4K20me1 increased dramatically during mitosis (Figure 2A and Figure S1). This is consistent with reports showing that in other organisms, H4K20me1 is present at high levels in mitosis and has a role in chromosome condensation [16]. At around the 30-cell stage, DPY-27 became localized to subregions of each nucleus that have been shown to be the X chromosomes [14] (Figure 2B, 2F). At this time, H4K20me1 was still distributed uniformly in the nucleus (Figure 2B, 2F). In embryos beginning the differentiation stage, H4K20me1 staining began to concentrate on the X chromosomes, as evidenced by colocalization with DPY-27, and this pattern became widespread in somatic cells throughout hermaphrodite development (Figure 2D, 2E, 2G; Figure S2A). Therefore, localization of the DCC to the X chromosome precedes enrichment of H4K20me1.

### H4K20me1 is reduced on the X in the germ line during meiotic silencing

In the germ line, the DCC does not localize to the X chromosome [10]. The X chromosome is largely silent, and the silent state is mediated by a mechanism independent from somatic dosage compensation [10], [17], [18], [19]. We observed a distinct and dynamic pattern of H4K20me1 staining in the adult germ line. H4K20me1 levels were highest in the distal mitotic region, and decreased as nuclei entered meiosis (Figure S3A). Early meiotic nuclei entering the transition zone showed similar H4K20me1 levels on X and autosomes (Figure S3B). As nuclei progressed through the transition zone into early pachytene, H4K20me1 levels were much lower on the X chromosome than on autosomes (Figure S3B). The X chromosome is silent during early and mid-pachytene [18]. Later in meiotic prophase, H4K20me1 levels increased on the X, coincident with activation of X-linked gene expression (Figure S3B; [18]). In contrast to the enrichment of H4K20me1 on the X chromosome in somatic nuclei, levels of H4K20me1 in late meiotic prophase were similar on the X chromosome and autosomes (Figure S3B). To summarize, H4K20me1 shows a dynamic localization pattern on germline chromatin, with two characteristics of localization shared with somatic chromatin. First, enrichment of H4K20me1 is observed on mitotic chromosomes. Second, in meiotic nuclei, H4K20me1 is associated with chromatin that has active gene expression.

### H4K20me1 enrichment on the X depends on DCC function

**Figure 3:**
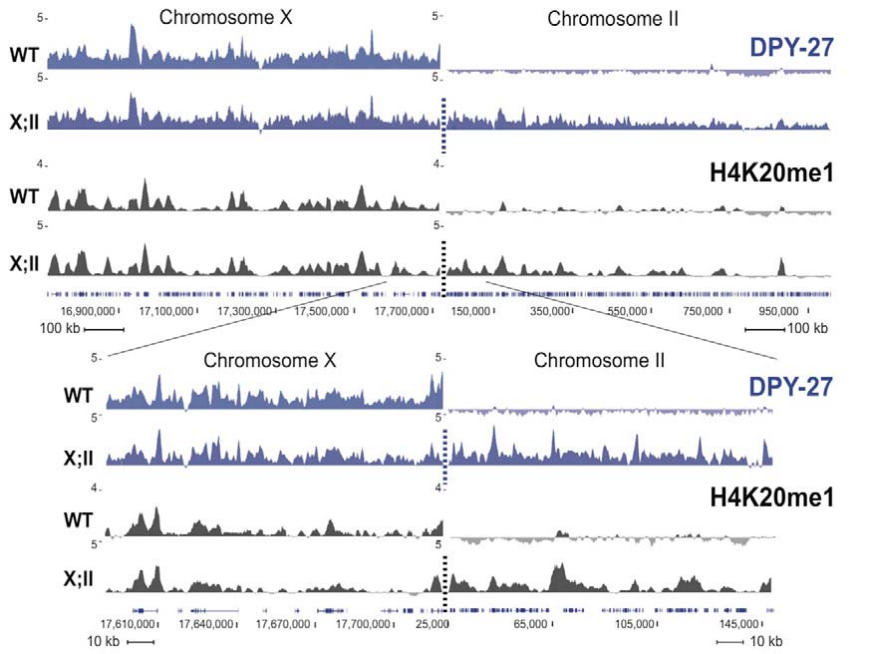
H4K20me1 ectopically spreads onto the autosomal region of an X;II fusion chromosome. H4K20me1 ChIPs were performed in wild-type (WT) and YPT41 mixed stage embryos. YPT41 carries a fusion of the right end of X to the left end of chromosome II. The fusion site is indicated with a dashed line. DPY-27 ChIP-chip and H4K20me1 ChIP-seq enrichment are shown in blue and black, respectively. For each track, the average of two independent biological replicates is shown as z-scores (standardized log_2_ ratios of ChIP/Input signals). Genome browser views of ∼2 Mb and ∼200 kb spanning the fusion site are shown on the top and below, respectively.

To test whether H4K20me1 enrichment on the X chromosome depends on the DCC, we carried out H4K20me1 ChIP-chip experiments using extracts from *dpy-21* mutant L3 larvae, which are deficient in dosage compensation [12]. Strikingly, H4K20me1 enrichment on the X relative to autosomes was abolished in *dpy-21* mutants (Figure 1B–1F), indicating that the DCC facilitates enrichment of H4K20me1 on the X. To further investigate this link, we asked whether the DCC could increase H4K20me1 levels at ectopic sites. Previous studies showed that DCC proteins bind recruiting elements on the X and then spread to neighboring regions that lack recruiting sites [6], [20]. Spreading is observed in chromosomal fusions between the X chromosome (which contains recruiting sites) and an autosome (which lacks recruiting sites; Figure 3). Using this assay, we observed spreading of H4K20me1 into the autosomal region flanking the site of the fusion, similar to that seen for the DCC component DPY-27 (Figure 3). This is consistent with a direct role for the DCC in generating enrichment of H4K20me1 on the X.

**Figure 4:**
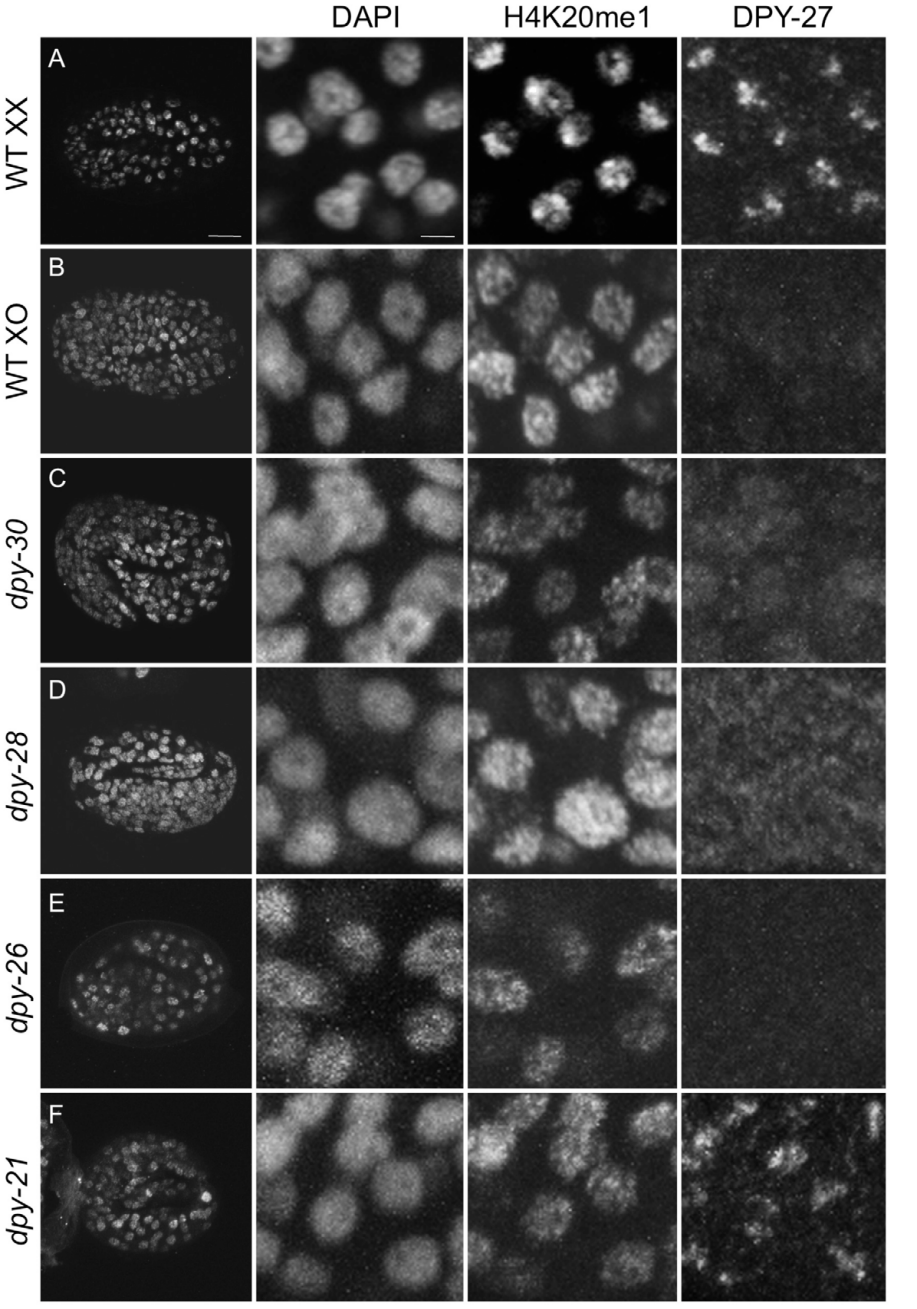
H4K20me1 enrichment to the X chromosome depends on dosage compensation. Three-fold embryos were stained with DAPI and antibodies to DPY-27 and H4K20me1. (A) Wild-type (WT) XX hermaphrodite, (B) *him-8* XO male, (C) *dpy-30(y228)* (D) *dpy-28(y1)*, (E) *dpy-26(n199)*, and (F) *dpy-21(e428)* hermaphrodites. Left panel shows whole embryos, right panels show enlargements of nuclei. Foci of H4K20me1 X-chromosome enrichment are absent in males and dosage compensation mutants. Scale bars represent 10 um (left column) and 2 um (enlargements). Monoclonal antibody 15F11 antibody was used to detect H4K20me1.

To better define the timing and requirement for dosage compensation in H4K20me1 enrichment on the X, we performed immunofluorescence experiments on dosage compensation mutant embryos. H4K20me1 was not enriched on the X chromosome in any dosage compensation mutant tested (*dpy-21*, *dpy-26*, *dpy-28*, and *dpy-30;* Figure 4 and Figure S4). Instead, H4K20me1 was evenly distributed on all chromosomes. We next analyzed XO males, which do not undergo dosage compensation and found no H4K20me1 enrichment on the single X of XO embryos or XO adult somatic nuclei (Figure 4B, Figures S2B and S4B). We also observed no H4K20me1 enrichment to X in adult gut nuclei of DCC mutants, similar to [21] (Figure S2). These results indicate that the DCC is required for the X-chromosome enrichment of H4K20me1.

### SET-1 and SET-4 enzymes generate H4K20me1 and H4K20me2/3, respectively

To determine whether H4K20me1 functions in dosage compensation, we sought to identify the enzymes responsible for methylation of H4K20. In other organisms, PR-Set7/SETD8 catalyzes monomethylation of H4K20 and Suv4-20 catalyzes di- and trimethylation of H4K20 [22], [23], [24]. The *C. elegans* orthologs of these proteins are SET-1 (PR-Set7/SETD8) and SET-4 (Suv4-20). Deletion mutants for both genes are available: *set-1(tm1821)* homozygotes develop into sterile adults (S. Mitani, pers. comm.), whereas *set-4(n4600)* homozygotes are viable and fertile [25].

**Figure 5:**
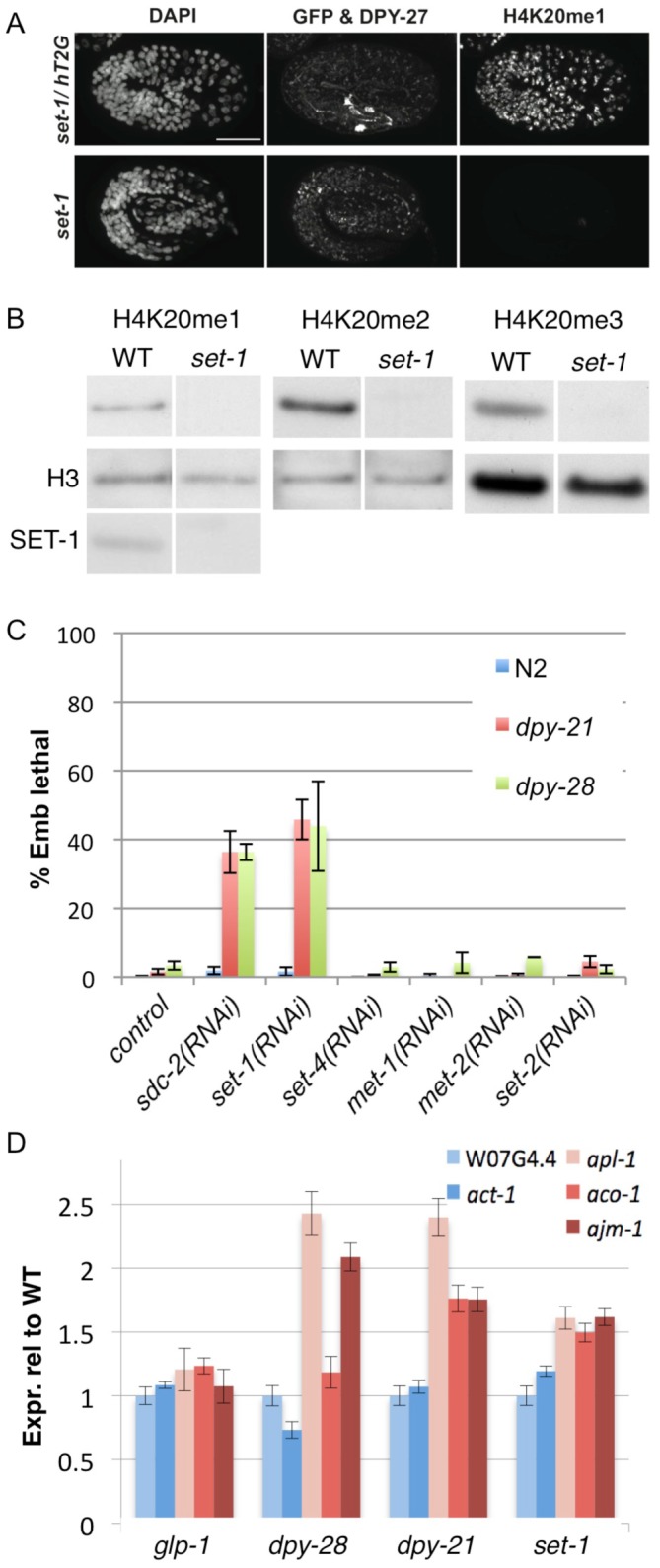
The H4K20me1 methyltransferase SET-1 genetically interacts with dosage compensation mutants and is required for repression of X-linked gene expression. (A) Embryos were stained with DAPI and antibodies to DPY-27 and H4K20me1. In *set-1/hT2G* embryos (identified by pharyngeal GFP signal from the *hT2G* balancer chromosome), DPY-27 and H4K20me1 are enriched on the X chromosome. In homozygous *set-1* mutant embryos, DPY-27 is enriched on the X, but H4K20me1 is undetectable. Scale bar represents 10 um. (B) Western blots of H4K20me1, H4K20me2, and H4K20me3 in wild-type (WT) and *set-1(tm1821)* mutant adults. The loading control is histone H3. SET-1 protein is not detected in *set-1* mutant extract. (C) Tests of genetic interactions with dosage compensation mutants. Shown is % embryonic lethality of wild type (WT), *dpy-21(e428)* and *dpy-28(y1ts)* mutants after RNAi of the indicated gene. RNAi was performed by feeding as described in Methods. (D) Expression of autosomal genes in blue (W07G4.4 and act-1) and X-linked genes in red (*aco-1*, *ajm-1*, and *apl-1*) in the indicated mutants relative to wild-type. Expression levels are normalized to autosomal gene W07G4.4. Error bars show 95% confidence intervals.

Among embryos from fertile *set-1/balancer* mothers, heterozygous *set-1/balancer* embryos contain robust H4K20me1 while *set-1* homozygous mutant embryos lack detectable H4K20me1 staining (Figure 5A). Furthermore, by western blot analysis, *set-1* mutant adult extracts lack detectable H4K20me1, as well as H4K20me2 and H4K20me3 (Figure 5B). We conclude that SET-1 is the major histone methyltransferase required for generation of H4K20me1.

**Figure 6:**
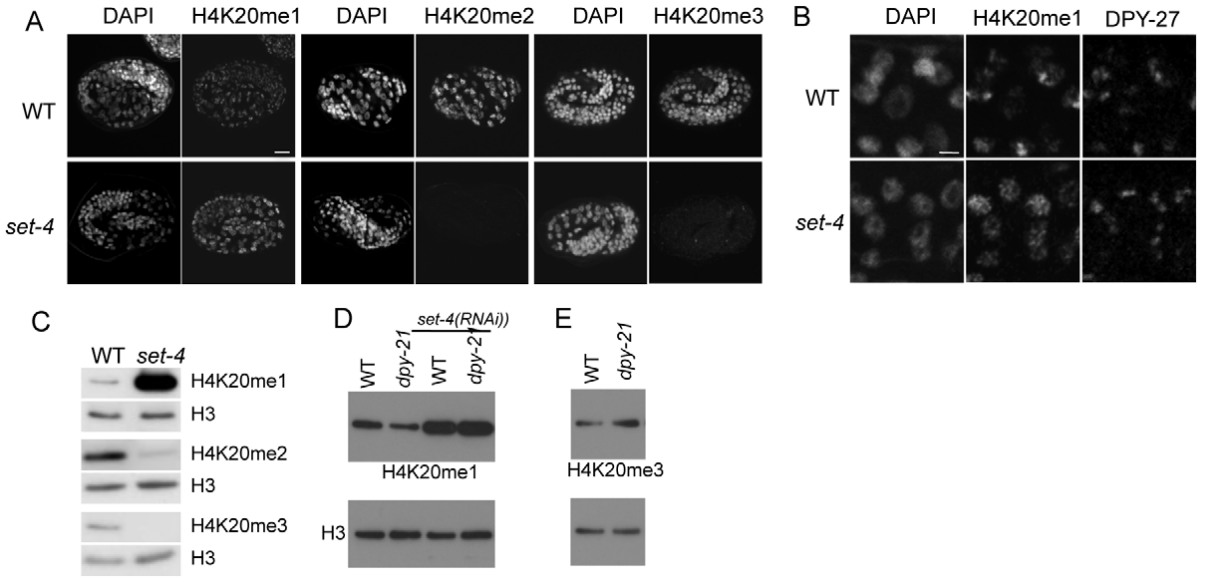
The SET-4 histone methyltransferase is necessary to generate H4K20me2 and H4K20me3. (A) Wild-type (WT) and *set-4(n4600)* mutant embryos were stained for DNA (DAPI), and H4K20me1, H4K20me2, or H4K20me3. H420me1 levels are higher and H4K20me2 and H4K20me3 levels strongly reduced in *set-4* mutant embryos. Scale bar, 10 um. (B) Enlargements of nuclei in wild-type and *set-4(n4600)* mutant embryos stained for DNA (DAPI), H4K20me1, and DPY-27. In *set-4* embryos, H4K20me1 does not show enrichment in DPY-27 positive regions. Confocal settings for H4K20me1 in *set-4* were reduced to allow comparison with wild-type images. Scale bar, 2 um. (C) Western blots of extracts made from wild-type and *set-4* adults, probed for H4K20me1, H420me2, or H4K20me3. Histone H3 is a loading control. Compared to wild type, *set-4* mutants have increased H4K20me1 levels, and reduced H4K20me2 and H4K20me3 levels. (D, E) Western blots of H4K20me1 (D) and H4K20me3 (E) in wild-type and *dpy-21* L3 mutant extracts. Lower panels show H3 loading controls. (D) H4K20me1 abundance is lower in *dpy-21* than in wild type. (E) H4K20me3 abundance is higher in *dpy-21* mutant L3 extract than in wild type. In (D) RNAi of *set-4* was additionally performed. H4K20me1 abundance is elevated in both wild-type and *dpy-21* after depletion of *set-4*. The following primary antibodies were used: H4K20me1 (Diagenode SN-147), H420me2 (Kimura 2E2), H4K20me3 (Abcam ab78517).

*C. elegans* SET-4 appears to be the major histone methyltransferase for converting H4K20me1 to H4K20me2 and H4K20me3. In *set-4(n4600)* homozygotes, we observed strongly reduced levels of H4K20me2 and H4K20me3 and increased levels of H4K20me1 compared to wild type, both by western blot and immunofluorescence analyses (Figure 6). In summary, our results show that SET-1 generates H4K20me1 and SET-4 converts H4K20me1 to H4K20me2 and H4K20me3, consistent with a recent report [21]. We note that DPY-27 localization appears normal in *set-1* and *set-4* mutants (Figure 5A and Figure 6B), suggesting that H4K20 methylations are not important for DCC recruitment.

### The X chromosome has lower levels of H4K20me2/3 than autosomes

In contrast to H4K20me1, the nuclear distributions of H4K20me2 and H4K20me3 are relatively uniform. To examine the levels of H4K20me2 and H4K20me3 more closely, we. co-stained late stage embryos for DPY-27 to mark the X chromosome. We observed that H4K20me2 and H4K20me3 were depleted on the X chromosome relative to autosomes (Figure S5). These differences were not observed in the DCC mutant *dpy-21* (Figure S5). We investigated the distribution of H4K20me3 at higher resolution using ChIP. Similar to other organisms (e.g. [26]), H4K20me3 is found at higher levels on inactive genes (bottom 20%) than on active genes (top 20%) (Figure 1C). However, overall levels are lower on the X chromosome compared to the autosomes, consistent with immunofluorescence results (Figure 1C–1E and Figure S5). We conclude that in hermaphrodites, the X chromosome has higher levels of H4K20me1 and lower levels of H4K20me2 and H4K20me3 relative to autosomes.

### Regulation of the SET-4 H4K20me2/3 methyltransferase contributes to X-chromosome enrichment of H4K20me1

In principle, these patterns could be generated through differential activity of SET-1 or SET-4 on X versus autosomes. For example, SET-1 might be more active in generating H4K20me1 on the X chromosome compared to autosomes, or SET-4 could be more active in converting H4K20me1 to H4K20me2 or H4K20me3 on autosomes compared to the X. To distinguish between these possibilities, we investigated H4K20me1 patterns in *set-4* mutant embryos. We observed that H4K20me1 levels in *set-4* mutant embryos were elevated and uniformly distributed in all nuclear regions, with no detectable X-chromosome enrichment (Figure 6B, 6C; similar to [21]). Similarly, ChIP experiments show that H4K20me1 is present at similar levels on the X chromosome and autosomes in *set-4* L3 mutant extracts (Figure S6). We conclude that the higher level of H4K20me1 on the X chromosome compared to the autosomes in wild type is achieved at least in part through higher SET-4 directed conversion of H4K20me1 to H4K20me2/3 on the autosomes.

If dosage compensation inhibits SET-4 from acting on the X chromosome, then dosage compensation mutants would be expected to have increased activity of SET-4 on the X, and as a result, reduced H4H20me1 and increased H4K20me3 levels compared to wild-type. To test this hypothesis, we carried out western blot analyses using dosage compensation mutant *dpy-21* and *sdc-1* L3 extracts. We found that the level of H4K20me1 is reduced in these mutants compared to wild type (Figure 6D and data not shown). Reduced H4K20me1 is due to inappropriate SET-4 activity, because levels are greatly increased in *dpy-21; set-4(RNAi)* or *sdc-1; set-4(RNAi)* animals (Figure 6D and data not shown). Furthermore, we found that the reduction in H4K20me1 in *dpy-21* mutants is accompanied by an increase in the level of H4K20me3, as expected if SET-4 has increased activity (Figure 6E). These results support the hypothesis that dosage compensation inhibits SET-4 activity on the X chromosome, leading to relatively high H4K20me1 levels and relatively low H4K20me3 levels on X.

### The SET-1 H4K20me1 methyltransferase is important for dosage compensation

We reasoned that if H4K20me1 is important for dosage compensation, then reducing H4K20me1 levels should impair this process. To test this hypothesis, we depleted SET-1 in two mutant backgrounds partially compromised for dosage compensation: null *dpy-21(e428)* mutants, which are viable, and a weak temperature-sensitive allele of *dpy-28(y1ts)*, which shows low embryonic lethality at the permissive temperature [12], [27]. A complete lack of dosage compensation in XX hermaphrodites results in 100% embryonic lethality [28]. Therefore, further perturbing the dosage compensation process in the sensitized backgrounds should cause an increase in embryonic lethality. We first validated the assay by testing whether knockdown of three different DCC subunits causes synthetic lethality in the two dosage compensation mutant backgrounds described above. As expected, RNAi of *sdc-2*, *dpy-27*, or *dpy-30* resulted in greatly increased lethality in *dpy-21* and *dpy-28(ts)* mutant backgrounds compared to wild type (Figure 5C and data not shown). Using this assay we found that RNAi of *set-1* induced synthetic lethality in *dpy-21* and *dpy-28(ts)* mutant backgrounds, elevating embryonic lethality from 2% in a wild-type background to 46% and 44% in *dpy-21* and *dpy-28(ts)*, respectively (Figure 5C). RNAi of *set-4* or of three other histone methyltransferases (*met-1*, *met-2*, *set-2)* had no effect (Figure 5C). The specific synthetic genetic interactions between *set-1* and dosage compensation mutants support the view that H4K20me1 is important for dosage compensation.

In *C. elegans*, the DCC represses dosage-compensated genes on the X by approximately 2-fold. To test directly the role of H4K20me1 in this repression, we compared the expression of X-linked dosage-compensated genes to non-compensated autosomal genes in wild-type and *set-1* mutant animals. Because *set-1* homozygous mutants are sterile and the X chromosome is not dosage compensated in the germ line, we focused on genes expressed in the soma. In order to control for possible germline effects, we performed analyses in *glp-1* mutant animals, which lack a germ line. Finally, as some X-linked genes are not dosage compensated, we chose X-linked genes that are upregulated in dosage compensation mutants *dpy-21* or *dpy-28*. As expected, we found that the expression of three X-linked genes (*aco-1*, *ajm-1*, and *apl-1*) relative to two autosomal genes (W07G4.4 and *act-1*) is unchanged in *glp-1* mutants and significantly increased in *dpy-21* and/or *dpy-28* mutants (Figure 5D). Similar to the DCC mutants, we observed a significant increase in the expression of dosage compensated X-linked genes relative to autosomal genes in the *set-1* mutant (Figure 5D). These results show that H4K20 monomethylation mediated by SET-1 has a role in repression of dosage-compensated X-linked genes.

## Discussion

Downregulation of X-linked gene expression during *C. elegans* dosage compensation allows study of gene expression mechanisms that act over large chromosomal regions. Previous studies have identified a condensin-like complex and other chromatin-associated proteins required for this process, but the mechanism by which these proteins lower X-linked gene transcription is not known. Here we show that the DCC generates X-linked enrichment of the post-transcriptional histone modification H4K20me1 and that this modification is important for dosage compensation.

As we observed here for *C. elegans*, H4K20me1 in other organisms is enriched on gene regions and its level is positively correlated with gene expression [29]. Although H4K20me1 levels are highest on actively transcribed genes, functional experiments in vertebrates and *Drosophila* point to a repressive role in gene expression. Knockdown of the H4K20me1 methyltransferase Pr-Set7 in human cells caused a two-fold upregulation of genes normally harbouring H4K20me1 [29], and mutation of Pr-Set7 in *Drosophila* leads to position effect variegation, a hallmark of genes required for heterochromatic gene repression [30]. H4K20me1 is also associated with the inactive X chromosome during X inactivation in vertebrates [31]. Therefore, the high levels of H4K20me1 we observed on the *C. elegans* X chromosome are consistent with a role in dosage compensation-mediated repression of gene expression.

What is the mechanism that leads to higher H4K20me1 levels and lower H4K20me3 levels on the hermaphrodite X chromosomes relative to autosomes? Our results suggest that this is achieved at least in part through DCC inhibition of SET-4 activity on X. Lower conversion of H4K20me1 to H4K20me2/3 by SET-4 on the X chromosome would lead to relatively higher H4K20me1 levels and lower H4K20me2/3 levels there. Several observations support this model. First, dosage compensation mutants show lower overall H4K20me1 levels and higher H4K20me3 levels compared to wild type. Second, the lower overall H4K20me1 level in DCC mutants is due to inappropriate SET-4 activity, supporting the idea that active dosage compensation inhibits SET-4 activity. Third, the difference in H4K20me1 levels on the X versus the autosomes is abolished in *set-4* mutants, indicating a role for SET-4 in generating the asymmetry. We propose that a component of the DCC prevents SET-4 from acting on the X chromosome, leading to maintenance of H4K20me1 on X, whereas H4K20me1 is preferentially converted to H4K20me2/3 on the autosomes. Because H4K20me1 levels are similar on X and autosomes in *set-4* mutants, SET-1 might be equally active in generating H4K20me1 on all chromosomes.

Our results suggest that of the three H4K20 methylation states, H4K20me1 is the key modification for dosage compensation. Whereas loss of dosage compensation leads to lethality of XX embryos, *set-4* null mutants, which have strongly reduced levels of H4K20me2 and H4K20me3, are viable, and RNAi of *set-4* does not enhance lethality of DCC mutants. This suggests that these modifications are not necessary for dosage compensation. In contrast, RNAi depletion of maternal and zygotic *set-1* leads to loss of H4K20me1 and embryonic lethality, *set-1* genetically interacts with dosage compensation mutants, and *set-1* mutants show upregulation of X-linked gene expression.

When does H4K20me1 function in dosage compensation? The DCC is recruited to the X chromosome around the 30-cell stage whereas X-chromosome enrichment of H4K20me1 occurs several hours later. This difference in timing suggests that there might be two separable aspects of dosage compensation during embryogenesis, for example initiation and maintenance. Although the DCC is recruited to the X in early embryogenesis, it is not yet known when repression of X-linked gene expression is initiated. The DCC might be active immediately after recruitment or might become active later in embryogenesis. Furthermore, although H4K20me1 becomes highly enriched on X in late embryogenesis, it is possible that a basal level on the X chromosome is functional earlier. Because the DCC component DPY-27 shows apparently normal localization to the X chromosome in the absence of SET-1 or SET-4, H4K20me1 does not appear to be a recruitment signal for the DCC. Instead, H4K20me1 may be important for the function of the DCC in downregulating gene expression. Key future questions to address are when during embryogenesis X-linked gene expression is initially downregulated, and when H4K20me1 function is necessary.

Regulation of histone modification levels also occurs during dosage compensation in other organisms. For example, in Drosophila, where gene expression on the single X chromosome in males is upregulated two-fold to match that of the two X chromosomes in females, dosage compensation acts to increase H4K16ac levels on the single male X. In addition, the inactive X chromosome of female mammals displays high levels of several histone modifications, including H4K20me1 [31]. H4K20me1 enrichment correlates with Xist expression, is independent of transcriptional silencing, and marks the early steps of X inactivation [31].

In addition to the strong enrichment of H4K20me1 on the X chromosome in *C. elegans*, Liu et al. showed that several marks of gene activity, including H4K16ac, were lower on X linked genes than on autosomal genes [15]. Using immunofluorescence assays on gut nuclei, a recent report by Wells et al. showed that the X/A difference in H4K16ac levels depends on dosage compensation and on *sir-2.1*, a putative H4K16 deacetylase [21]. It is not clear if H4K16Ac plays a role in dosage compensation as depletion of *sir-2.1* did not genetically interact with a DC mutant. The enzyme that generates H4K16Ac is not yet known.

Using immunofluorescence assays, Wells et al. also observed that H4K20me1 enrichment on X is dependent on dosage compensation, and on SET-1 and SET-4 [21]. Our immunofluorescence results are broadly similar, and our ChIP experiments give a higher resolution view, strengthening these conclusions. In support of a role for methylation of H4K20 in dosage compensation, Wells et al. observed that simultaneous reduction of *set-1* and *set-4* by RNAi could rescue mutant males that normally die due to active dosage compensation. However, the H4K20 methylation state was not determined after simultaneous depletion of *set-1* and *set-4*, so the specific alteration of methylation of H4K20 that caused rescue is not known. The reported X/A differences in H4K16ac levels also depended on *set-1* and *set-4*, suggesting that H4K16Ac might be regulated by H4K20 methylation state. Although the exact mechanisms of dosage compensation vary, studies in different organisms suggest that global regulation of H4K16ac and H4K20me1 levels might be a conserved feature of these chromosome-wide gene regulation mechanisms.

Our results indicate that H4K20me1 is important for repression of X-linked gene expression. How might H4K20me1 function in transcriptional repression? Several links in the literature suggest roles for H4K20me1 in chromatin compaction. For example, the Malignant-Brain-Tumor (MBT) domains of human L3MBTL1 compact nucleosomal arrays by recognizing mono and dimethylation of H4K20 and H1bK26 [32]. Furthermore, L3MBTL1 has transcription repressor activity that is enhanced by Pr-Set7, and its chromatin association depends on H4K20me1 [33], [34]. It is not yet known whether *C. elegans* MBT repeat proteins LIN-61 or MBTR-1 are involved in dosage compensation or bind H4K20me1.

H4K20me1 has also been shown to be important during mitosis. H4K20me1 levels are high on mitotic chromatin ([35], [36] and this study), and in mammalian cells inhibition of Pr-Set7 leads to defects in cell cycle progression [36], [37], [38], [39]. Although the function of H420me1 in cell cycle progression is not yet understood, a key aspect of the loss of function phenotype is reduced chromosome compaction. Furthermore, a recent study demonstrated that two components of condensin II, N-CAPD3 and N-CAPG2, can directly bind H4K20me1 [35]. This raises the exciting possibility that condensin I^DC^ might function to compact chromatin through binding H4K20me1. Increased compaction of the X chromosome relative to the autosomes might reduce access by RNA polymerase, leading to lower X-linked gene expression. Consistent with this idea, DCC mutants were recently shown to have increased RNA polymerase II levels on the X [13]. We propose that condensin complexes and H4K20me1 might be intimately linked in diverse chromatin-regulating events.

## Methods

### Worm culture and strains

The following strains were used and cultured using standard methods (Brenner, 1974): TY0621 [*yDp1*(IV,V:f); *dpy-26(n199) unc-30(e191)* IV], DR1410 [*dpy-27(y56)/qC1* III], TY1621 [*unc-49(e382) dpy-28(y1ts)* III], TY148 [*dpy-28(y1ts)* III], CB428 [*dpy-21(e428)* V], KK0423 [*dpy-21(e428) par-4(it57ts)* V], TY1936 [*dpy-30(y228)* V/*nT1[unc-?(n754) let-?*](IV;V)], SS1075 [*set-1(tm1821)/hT2G(qIs48) I;III*], JA1574 [*set-1(tm1821)/hT2G(qIs48) I;III*], MT14911 [*set-4(n4600*], YPT41 [X;II].

### Chromatin immunoprecipitation analyses

Late embryo (LE) extracts were prepared by growing wild-type N2 adult worms from synchronized L1s in standard S-basal medium with shaking. Worms were fed HB101 E. coli and grown at 20°C until they were gravid, approximately 70 hours. Embryos were obtained by dissolving adult worms with bleach, and then the embryos were aged by incubating in M9 media for 3.5 hrs at 20°C with gentle rocking. The embryos were washed once with PBS and flash frozen in liquid nitrogen, then processed for ChIP as in [40]. Wild-type, *dpy-21(e428)*, and *set-4(n4600)* L3 and *fem-2* YA animals were grown and ChIPs performed as in [41] except that DNA was sonicated to a size range of 200–400 bp. EE and L3 in Figure 1 are from [15] (Abcam ab9051; lot 104513). H4K20me1 ChIP conditions were: LE (1 mg extract and 5 ul Diagenode SN-147), *fem-2* young adults (1.25 mg extract and 3 ug Abcam ab9051 lot 104513), *dpy-21* L3s (500 ug extract and 2 ug Abcam ab9051 lot 104513), WT and *set-4* L3s in Figure S6 (500 ug extract and 2 ug Abcam ab9051 lot 602259), WT L3 in Figure S7 (500 ug extract and 5 ul Diagenode SN-147). The Diagenode SN-147 and Abcam ab9051 antibodies give concordant patterns by ChIP in L3 extracts (Figure S7A, S7B). H4K20me3 ChIP conditions: 500 ug L3 extract and XX Abcam 78517 lot 827718. Antibodies used for ChIP, western blot, or immunofluorescence were tested for specificity to histone peptide tails using dot blots ([42] and http://compbio.med.harvard.edu/antibodies/).

Early Embryo, Late Embryo, L3, and *dpy-21* L3 H4K20me1 ChIPs and L3 H4K20me3 ChIPs were hybridized to a *C. elegans* full-genome tiled microarray (NimbleGen 2.1, Roche). For H4K20me1 ChIPs, log2 ratios of IP/Input were obtained and standardized so the autosomal signal had mean 0 and standard deviation 1. The H4K20me3 L3 ChIP dataset was processed similarly except that all chromosome regions were used. *fem-2* young adult (Figure 1) and WT and *set-4* L3 H4k20me1 ChIPs in Figure S6 were sequenced on the Illumina platform, aligned using BWA with default settings [43], normalized using BEADS [44], then converted to log2 ratios of BEADS scores (enrichment relative to input) and standardized so the mean of the autosomal signal was 0 and the standard deviation 1. Biological ChIP-chip and ChIP-seq replicates were averaged after standardization. Gene profile plots (Figure 1 and Figure S6) were generated by aligning genes at their TSS and TES (WS190/ce6). The genomic regions 1 kb upstream to 1 kb downstream from TSS and 1.5 kb upstream to 1 kb downstream from TES were divided into 50-bp bins. Genes were grouped into top 20% and bottom 20% expressed and autosomal and X-linked genes. Mean signals for each group of genes and each bin were plotted as well as the 95% confidence intervals of the mean (as error bars). Profile plots of intergenic regions were obtained by first identifying regions of at least 10 kb length without any annotation (WS190/ce6) (491 such regions on autosomes, 193 on chromosome X). The regions were aligned at their center and the genomic regions from 5 kb upstream to 5 kb downstream from the center were divided into 50 bp bins. Mean signals for the autosomal and X intergenic regions were plotted as well as the 95% confidence intervals of the means.

Boxplots (Figure 1 and Figure S7) were obtained after 1 kb median smoothing along the chromosomes of the respective standardized log2 ratios. Each box indicates the median with the center line and extends from the 25th to the 75th percentile of the standardized log2 ratios; whiskers extending from the box indicate the 2.5th and 97.5th percentiles.

Transcript data for the EE, LE, L3 and YA stages were obtained from the modENCODE DCC (http://intermine.modencode.org). The platform was a single color 4-plex Nimblegen expression array with 72,000 probes (three 60-mer oligo probes per gene). Quantile normalization [45] and the Robust Multichip Average (RMA) algorithm [46] were used to normalize and summarize the multiple probe values per gene to obtain one expression value per gene and sample.

### Western blotting

Sample buffer was added directly to worms, samples heated to 65°C for 10 minutes, sonicated for 15 minutes (30 sec in/30 sec out), incubated at 65°C for 5 min and finally boiled at 95°C for 5 min. Proteins were separated on 4–12% NuPage SDS pre-cast gels (Invitrogen). The following antibodies were used: anti-H4K20me1 (Abcam ab9051 at 1∶2000), anti-H4K20me2 (H. Kimura monoclonal antibody 2E2 at 1∶20,000), anti-H4K20me3 (Abcam ab78517 at 1∶500), anti-H3 (Active Motif 39163 at 1∶8000), and anti-SET-1 (SDI SDQ3895). JA1574 was used to obtain *set-1* homozygotes in Figure 5B.

### Immunostaining

Immunostaining of embryos and dissected intestines was done using methanol/acetone fixation as in [47] (Figure 2; Figure 5; Figure S1, S2, and S4) or using methanol fixation as in [48] (Figure 4, Figure 6, Figure S5). Germline immunofluorescence experiments (Figure S3) were carried out after dissecting worms in egg buffer containing 0.1% Tween 20 and fixation in 1% formaldehyde in egg buffer [49]. SS1075 was used for the IF experiment in Figure 5A. Primary antibodies used are indicated in the figure legends. Antibody concentrations used were: anti-H4K20me1 Kimura 1F11 (1∶40,000), anti-H4K20me1 Diagenode SN-147 (1∶100,000), anti-H4K20me1 Abcam ab9051 (1∶400 for Figure S1; 1∶5000 for Figure S3), anti-H4K20me2 Kimura 2E2 (1∶25,000), anti-H4K20me3 ab78517 (1∶200), anti-DPY-27 SDI SDQ3995 (1∶4000), anti-GFP Abcam ab290 (1∶6000), anti-H3S10p Kimura 10H12 (1∶1000), anti-HIM-8 (1∶250). Secondary antibodies were purchased from Jackson ImmunoResearch or Molecular Probes.

### qPCR quantification of gene expression

Total RNA was extracted from N2 (wild type), *dpy-21(e428)*, *dpy-28(y1)*, or *set-1(tm1821)* L3 worms grown at 25°C using TriPure (Roche). RNA was further purified using an RNeasy column (Qiagen). Reverse transcription was carried out using the Invitrogen SuperScript III First-Strand Synthesis System. Quantitative PCR was performed using primers specific to the target genes:

Chromosome V:

W07G4.4 F: GCAATCGCTCCAGCCGTTAACAAT; R: TCGTCCAGATGGAACGACAGATGA

*act-1* F: TGCAGAAGGAAATCACCGCTCTTG; R: AAGCACTTGCGGTGAACGATGGAT

Chromosome X:

*apl-1* F: ACGACGACGATGAGGATGATGCTT; R: TGAACTTCTCGGCTCCCTTTGGAT

*aco-1* F: CAAGATCAACCCAGTATGCCCAGT; R: ACCTGATGGACGATTCCAGATCCT

*ajm-1* F: TCGTCTTGATGAGATGGAACGCGA; R: AAGTTCTGCGTTACGTTGGGCTTG

Gene expression levels in each strain were normalized to the levels of the autosomal gene W07G4.4. Expression of each gene in wild-type N2 was then set to 1 and mutant expression levels expressed relative to N2.

### Genetic interaction tests

RNAi by feeding was performed on N2 (wild type), *dpy-21(e428)* and *dpy-28(y1)* animals as follows: 3–5 L3 larvae were placed on RNAi bacteria for three days at 15°C, transferred to new RNAi plates for 24 hours, transferred again after 24 hours, then removed. The progeny on the latter two plates were scored for embryonic lethality. We note that RNAi of *set-1* leads to embryonic lethality in the N2 (wild-type) background in embryos produced in the next 24 hours of RNAi feeding, or if RNAi is performed by injection (not shown). RNAi plates were prepared as in [50]. The following RNAi clones were used from [51], [52]: *sdc-2* (C35C5.1), *set-1* (T26A5.7), *set-2* (C26E6.9), *met-1* (C43E11.3), and *met-2* (R05D3.11). The *set-4* RNAi clone was made by PCR amplifying and cloning a portion of the *set-4* gene into RNAi feeding vector L4440. The primers used were: atacgaattcacaggtcggc and tgctactacgcttgtcgtcg. RNAi plasmids were in the HT115(DE3) bacterial strain, which was used as the control. All RNAi clones were verified by sequencing.

### Assaying ectopic spreading

H4K20me1 ChIPs were performed in mixed stage embryos from wild-type N2 normal karyotype strain (2 replicates) and in YPT41 X;II fusion strain (2 replicates) using methods described previously [53]. 1 mg of total embryo extract and 2 ug of H4K20me1 antibody (Abcam ab9051) were used per ChIP. The ChIP DNA was prepared for Illumina multiplex sequencing with slight modifications to the manufacturers protocol [54]. Briefly, sequencing libraries were prepared from half of the ChIP DNA and 10 ng of corresponding input DNA. NEB Klenow, T4 DNA polymerase and T4 PNK were used to repair ends at 20°C for 30 min. Exo(-) Klenow fragment and dATP was used to add adenosine at the 3′ ends for 1 hour at 37°C. DNA was ligated to multiplex adaptors (Illumina) and amplified by PCR, introducing the following indices: N2 H4K20me1 ChIP index #6 (GCCAAT), N2 Input index #12 (CTTGTA), YTP41 H4K20me1 ChIP replicate 1 index #2 (CGATGT), input index #3 (TTAGGC), and H4K20me1 ChIP replicate 2 index #1 (ATCACG), input index #5 (ACAGTG). DNA between 200–500 bp in size was gel purified. Multiplexed single-end sequencing was performed by GAIIx at the UNC high-throughput sequencing facility. The sequencing reads, obtained from the Illumina pipeline in fastq format, were aligned to the ce6 (WS190) version of the *C. elegans* genome with Bowtie [55], using default parameters. Each sequence read was extended to calculate coverage per base pair using MACS [56]. The coverage from the input data was subtracted from that of the ChIP data, and ChIP enrichment was standardized by z score transformation.

### Datasets

Accession numbers for datasets generated in this paper are listed in Table S1.

## Supporting Information

Figure S1 Nuclear abundance of H4K20me1 is cell cycle regulated. Wild-type embryos were stained with DAPI (blue) and antibodies to H3S10ph (red) and H4K20me1 (green). In early embryos, H4K20me1 levels are higher on condensed prometaphase and metaphase chromosomes, marked with the mitotic marker H3S10ph, than in interphase nuclei. Scale bar represents 10 um. Monoclonal antibody 10H12 was used to detect H4K20me1.
(PDF)

Figure S2 H4K20me1 enrichment on the X chromosome in adult somatic cells depends on dosage compensation and SET-1. Dissected adult tissues were stained as in Figure 2. In wild-type (WT) XX intestinal nuclei (A) DPY-27 and H4K20me1 co-localize on the X chromosomes. H4K20me1 is diffusely nuclear in intestinal cells of wild-type XO males (B) and XX animals defective in dosage compensation: (C) *dpy-21(e428)*, (D) *dpy-26(n199)*, and (E) *dpy-28(y1)*. Intestinal nuclei of *set-1* mutant animals (F) lack detectable H4K20me1. The brightness of H4K20me1 in panel F is over-exposed to show the lack of nuclear stain. Scale bar represents 10 um. Monoclonal antibody 15F11 was used to detect H4K20me1.
(PDF)

Figure S3 H4K20me1 exhibits dynamic localization in the germ line. (A) Dissected gonads from wild-type hermaphrodites were stained with DAPI and an antibody to H4K20me1. H4K20me1 levels are high in the distal region corresponding to premeiotic nuclei. Levels drop as nuclei move proximally and enter meiosis. (B) Gonads were stained with DAPI (red) and antibodies to H4K20me1 (green) and an X-chromosome binding protein HIM-8 (blue). H4K20me1 is present on the X in transition zone nuclei in early meiotic prophase. In mid-pachytene, H4K20me1 is significantly depleted from the X. In late pachytene nuclei, H4K20me1 relocalizes to the X. The Abcam ab9051 antibody was used to detect H4K20me1.
(PDF)

Figure S4 H4K20me1 enrichment on the X chromosome depends on dosage compensation. This figure resembles Figure 4, but shows results with a different antibody to H4K20me1. Three-fold embryos were stained with DAPI and antibodies to DPY-27 and H4K20me1. In wild-type XX embryos (A) DPY-27 and H4K20me1 are concentrated on the X chromosome. In XO embryos (B) DPY-27 is absent and H4K20me1 is diffusely nuclear. In XX embryos deficient in dosage compensation, *dpy-21(e428)* (C), *dpy-26(n199)* (D), and *dpy-28(y1)* (E), H4K20me1 is diffusely nuclear. Scale bars represent 10 um (A–E, left column) and 2 um (A–E, enlargements). The Diagenode SN-147 antibody was used to detect H4K20me1.
(PDF)

Figure S5 H4K20me2 and H4K20me3 show reduced staining on X chromosomes compared to autosomes. Wild-type (WT) and *dpy-21* mutant embryos stained for DNA (DAPI), DPY-27, and (A) H4K20me2 or (B) H4K20me3. The X chromosome, marked by DPY-27, shows lower staining of both modifications than other regions. In *dpy-21* mutants, this difference is not observed.
(PDF)

Figure S6 Enrichment of H4K20me1 on X is abolished in *set-4* mutants. (A) Genome browser tracks of H4K20me1 ChIP-seq signals across representative regions of chromosome I and chromosome X in wild-type (WT) and *set-4* mutant L3 larvae. Signal is displayed as z-scores (standardized log_2_ ratios of ChIP/Input signals). Enrichment of H4K20me1 on X is lost in *set-4* mutants. (B) Plots of H4K20me1 signal across the TSS (transcript start site) and TES (transcript end site) of genes on X (red) and autosomes (blue). Genes in the top 20% of expression (dark shades) and bottom 20% (light shades) are plotted separately. (C) Plots of H4K20me1 signal centered at intergenic regions at least 5kb from any annotated feature. (D) Box plots of overall H4K20me1 signals on each chromosome. Each box shows the median and extends from the 25^th^ to the 75^th^ percentile of the z-scores in the set; whiskers extending from the box indicate the 2.5^th^ and 97.5^th^ percentiles.
(PDF)

Figure S7 Consistency of different H4K20me1 antibodies in ChIP. (A) Correlation between ChIP-chip signals using antibodies to H4K20me1 from Abcam and Diagenode (r = 0.95). (B) Plots of H4K20me1 signal across the TSS (transcript start site) and TES (transcript end site) of genes on X (red) and autosomes (blue). Genes in the top 20% of expression (dark shades) and bottom 20% (light shades) are plotted separately. Plots generated using data from the two antibodies show similar patterns.
(PDF)

Table S1 ChIP dataset accession numbers. modENCODE or GEO accession numbers for datasets generated or used in the paper. modENCODE data can be obtained from http://data.modencode.org/ or modMine: http://intermine.modencode.org/; GEO data can be obtained from http://www.ncbi.nlm.nih.gov/geo/
(XLS)
