# Supplementary figures and images for: H4K20me1 Contributes to Downregulation of X-Linked Genes for C. elegans Dosage Compensation
Source: PLoS Genet. 2012 Sep 13;8(9):e1002933. doi: 10.1371/journal.pgen.1002933 (PMC3441679; doi:10.1371/journal.pgen.1002933)

Figure S1

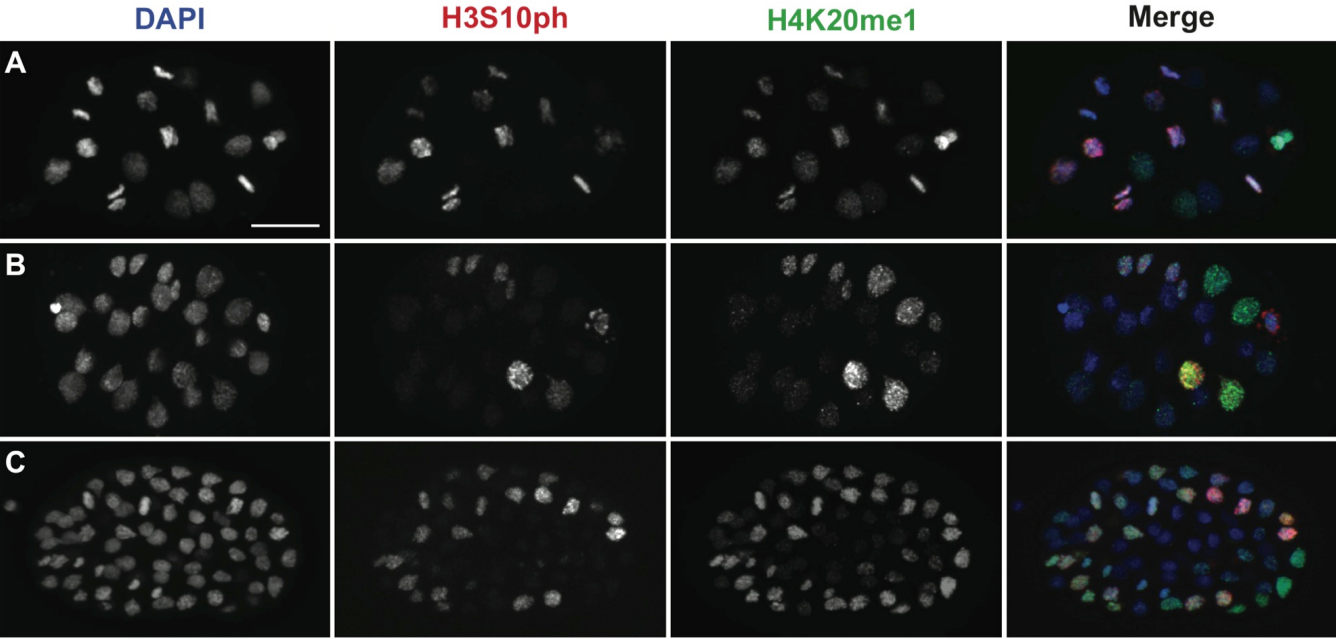

Supplement: Figure S1 — Nuclear abundance of H4K20me1 is cell cycle regulated. Wild-type embryos were stained with DAPI (blue) and antibodies to H3S10ph (red) and H4K20me1 (green). In early embryos, H4K20me1 levels are higher on condensed prometaphase and metaphase chromosomes, marked with the mitotic marker H3S10ph, than in interphase nuclei. Scale bar represents 10 um. Monoclonal antibody 10H12 was used to detect H4K20me1. (PDF) [file pgen.1002933.s001.pdf]

Figure S2

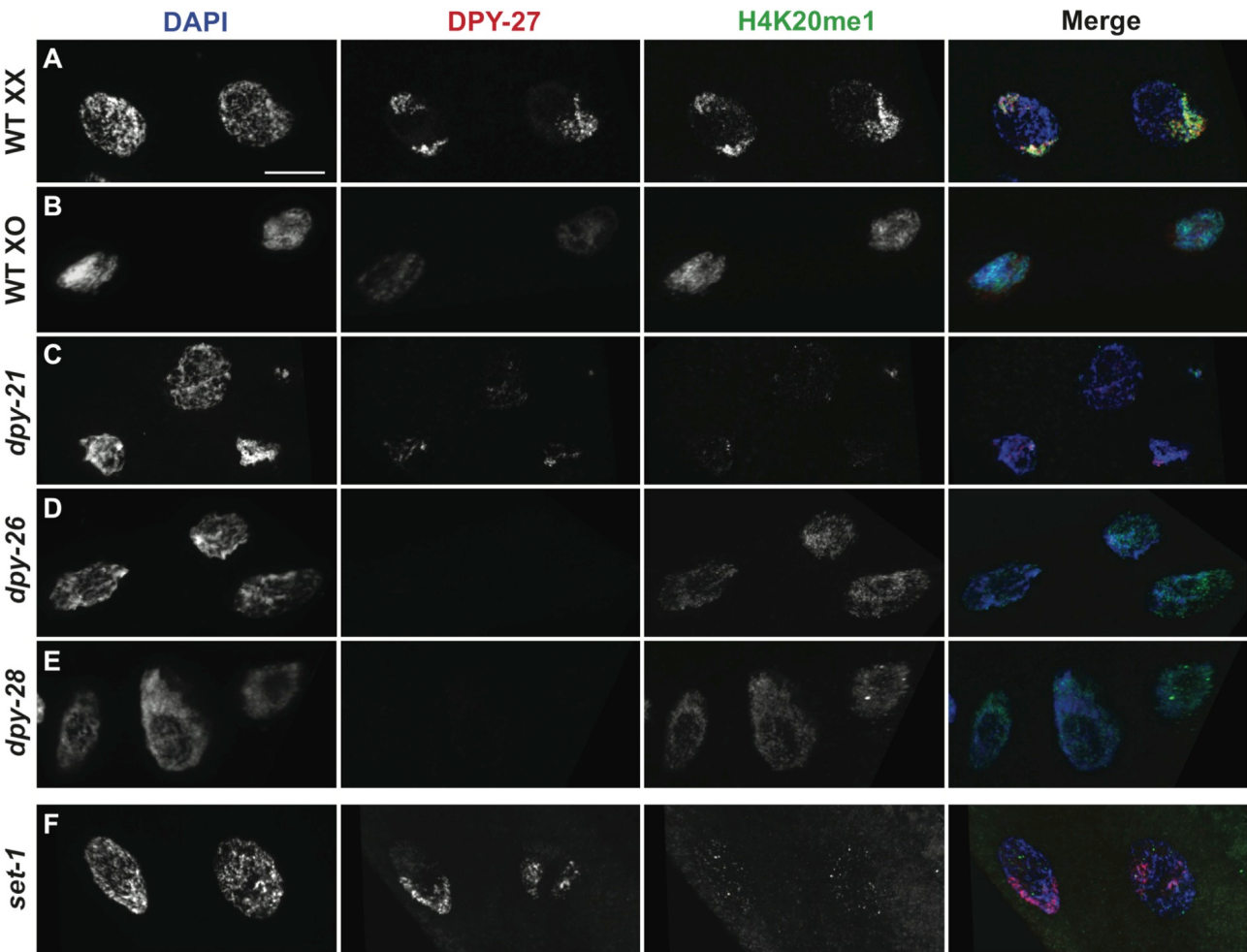

Supplement: Figure S2 — H4K20me1 enrichment on the X chromosome in adult somatic cells depends on dosage compensation and SET-1. Dissected adult tissues were stained as in Figure 2. In wild-type (WT) XX intestinal nuclei (A) DPY-27 and H4K20me1 co-localize on the X chromosomes. H4K20me1 is diffusely nuclear in intestinal cells of wild-type XO males (B) and XX animals defective in dosage compensation: (C) dpy-21(e428), (D) dpy-26(n199), and (E) dpy-28(y1). Intestinal nuclei of set-1 mutant animals (F) lack detectable H4K20me1. The brightness of H4K20me1 in panel F is over-exposed to show the lack of nuclear stain. Scale bar represents 10 um. Monoclonal antibody 15F11 was used to detect H4K20me1. (PDF) [file pgen.1002933.s002.pdf]

Figure S3

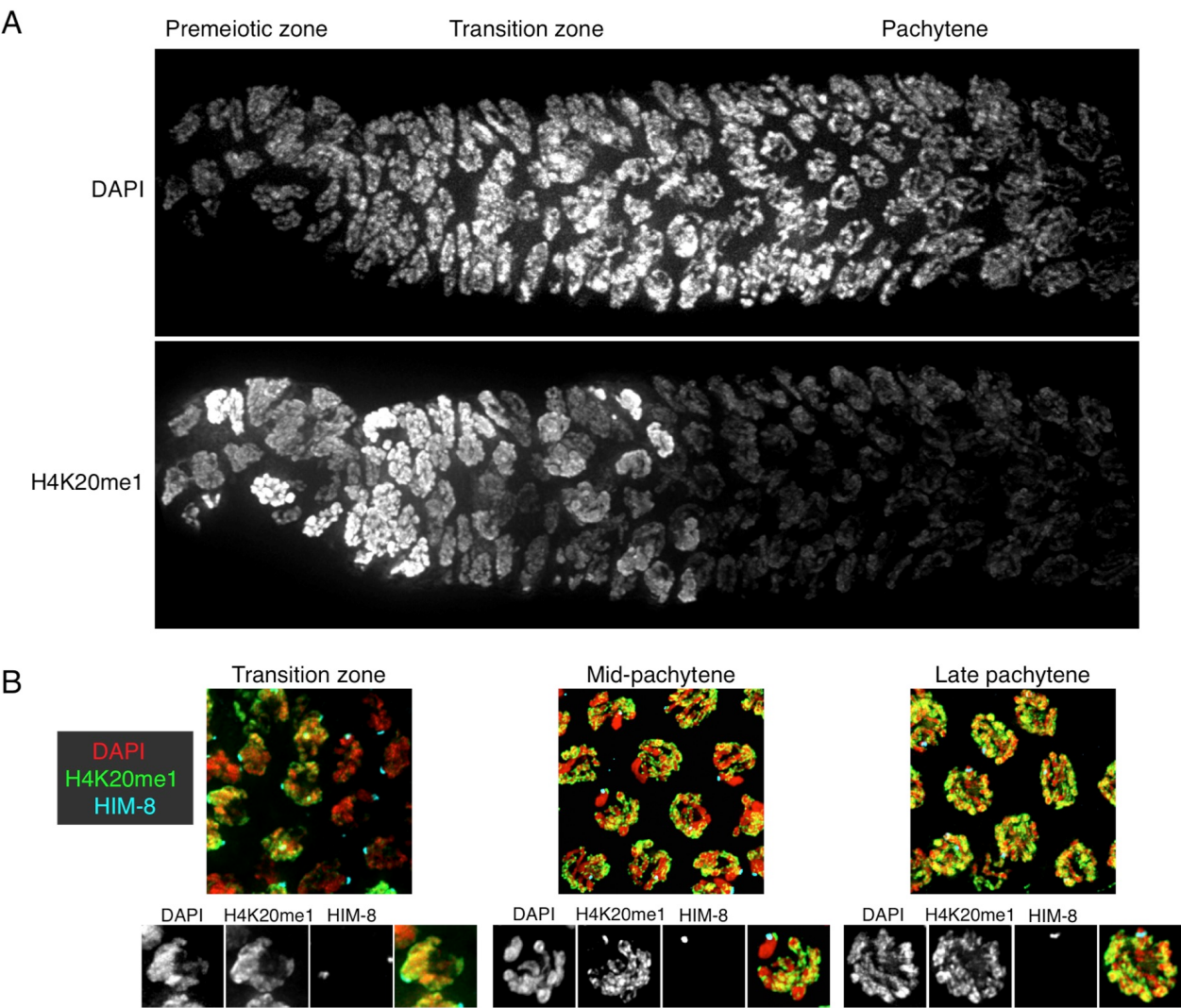

Supplement: Figure S3 — H4K20me1 exhibits dynamic localization in the germ line. (A) Dissected gonads from wild-type hermaphrodites were stained with DAPI and an antibody to H4K20me1. H4K20me1 levels are high in the distal region corresponding to premeiotic nuclei. Levels drop as nuclei move proximally and enter meiosis. (B) Gonads were stained with DAPI (red) and antibodies to H4K20me1 (green) and an X-chromosome binding protein HIM-8 (blue). H4K20me1 is present on the X in transition zone nuclei in early meiotic prophase. In mid-pachytene, H4K20me1 is significantly depleted from the X. In late pachytene nuclei, H4K20me1 relocalizes to the X. The Abcam ab9051 antibody was used to detect H4K20me1. (PDF) [file pgen.1002933.s003.pdf]

Figure S4

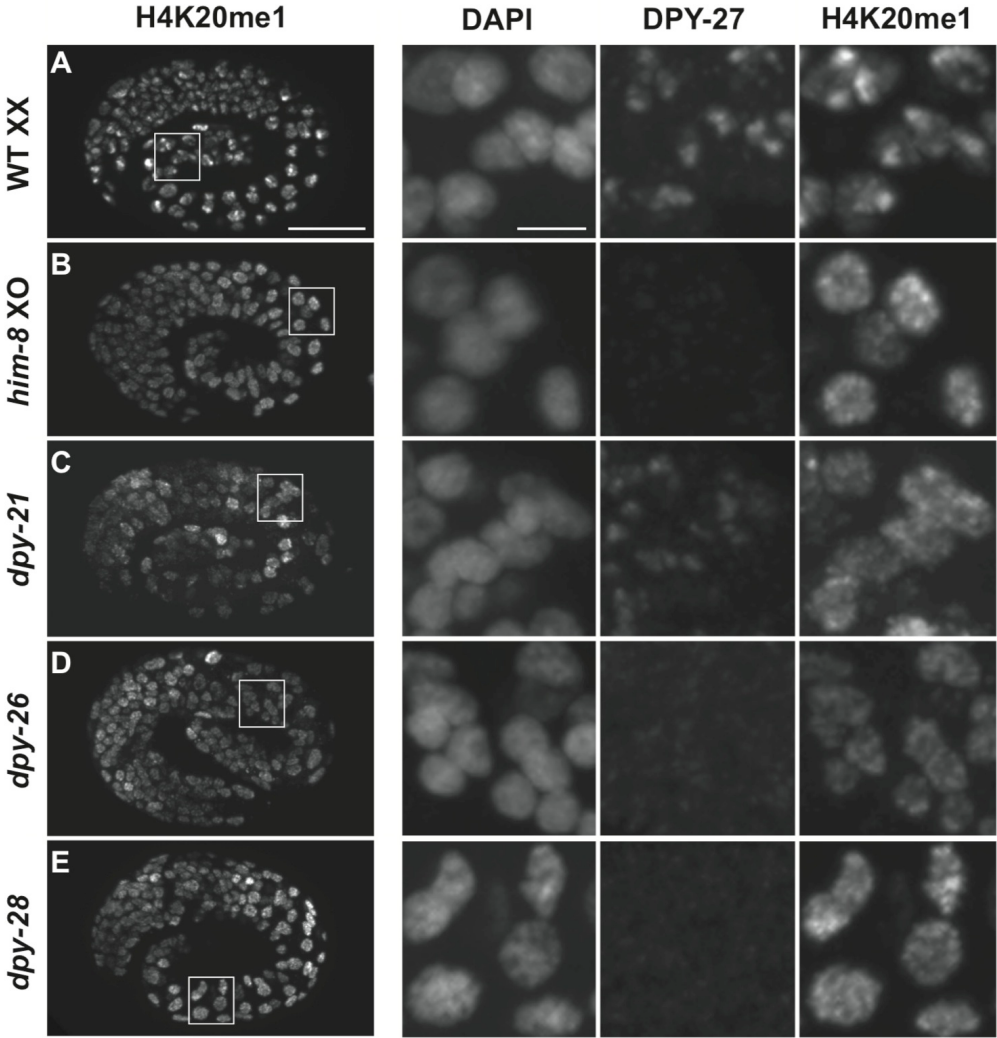

Supplement: Figure S4 — H4K20me1 enrichment on the X chromosome depends on dosage compensation. This figure resembles Figure 4, but shows results with a different antibody to H4K20me1. Three-fold embryos were stained with DAPI and antibodies to DPY-27 and H4K20me1. In wild-type XX embryos (A) DPY-27 and H4K20me1 are concentrated on the X chromosome. In XO embryos (B) DPY-27 is absent and H4K20me1 is diffusely nuclear. In XX embryos deficient in dosage compensation, dpy-21(e428) (C), dpy-26(n199) (D), and dpy-28(y1) (E), H4K20me1 is diffusely nuclear. Scale bars represent 10 um (A–E, left column) and 2 um (A–E, enlargements). The Diagenode SN-147 antibody was used to detect H4K20me1. (PDF) [file pgen.1002933.s004.pdf]

Figure S5

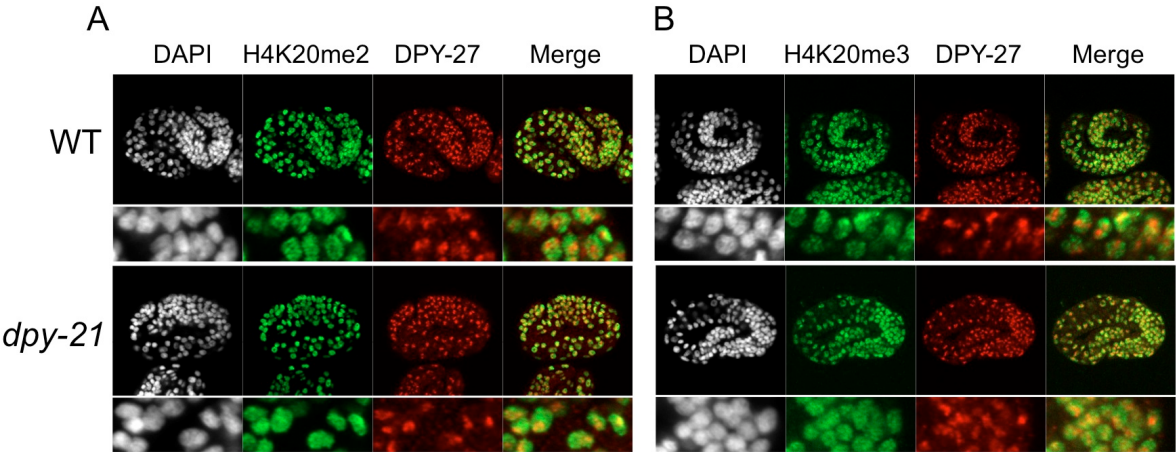

Supplement: Figure S5 — H4K20me2 and H4K20me3 show reduced staining on X chromosomes compared to autosomes. Wild-type (WT) and dpy-21 mutant embryos stained for DNA (DAPI), DPY-27, and (A) H4K20me2 or (B) H4K20me3. The X chromosome, marked by DPY-27, shows lower staining of both modifications than other regions. In dpy-21 mutants, this difference is not observed. (PDF) [file pgen.1002933.s005.pdf]

Figure S6

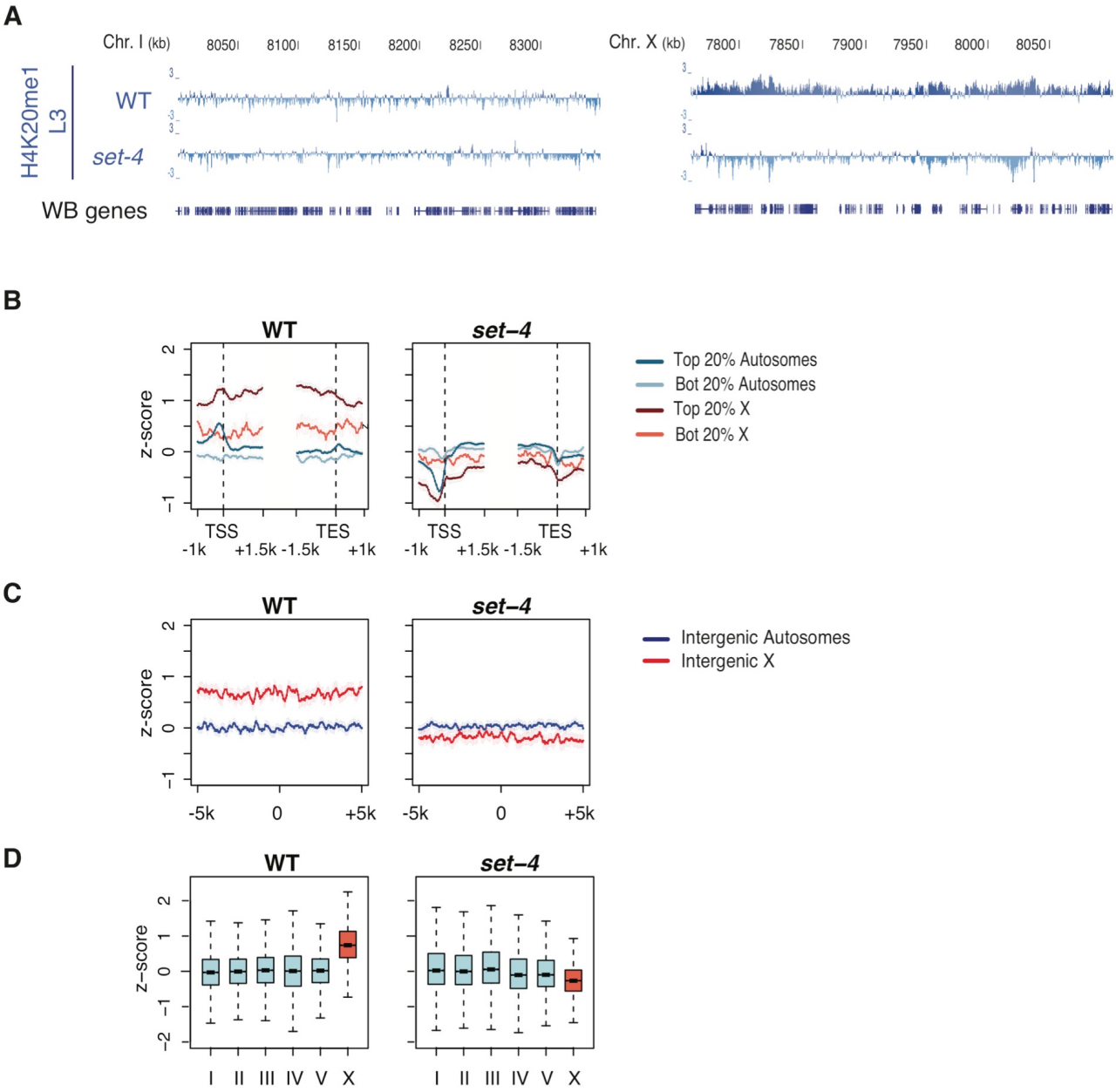

Supplement: Figure S6 — Enrichment of H4K20me1 on X is abolished in set-4 mutants. (A) Genome browser tracks of H4K20me1 ChIP-seq signals across representative regions of chromosome I and chromosome X in wild-type (WT) and set-4 mutant L3 larvae. Signal is displayed as z-scores (standardized log2 ratios of ChIP/Input signals). Enrichment of H4K20me1 on X is lost in set-4 mutants. (B) Plots of H4K20me1 signal across the TSS (transcript start site) and TES (transcript end site) of genes on X (red) and autosomes (blue). Genes in the top 20% of expression (dark shades) and bottom 20% (light shades) are plotted separately. (C) Plots of H4K20me1 signal centered at intergenic regions at least 5kb from any annotated feature. (D) Box plots of overall H4K20me1 signals on each chromosome. Each box shows the median and extends from the 25th to the 75th percentile of the z-scores in the set; whiskers extending from the box indicate the 2.5th and 97.5th percentiles. (PDF) [file pgen.1002933.s006.pdf]

Figure S7

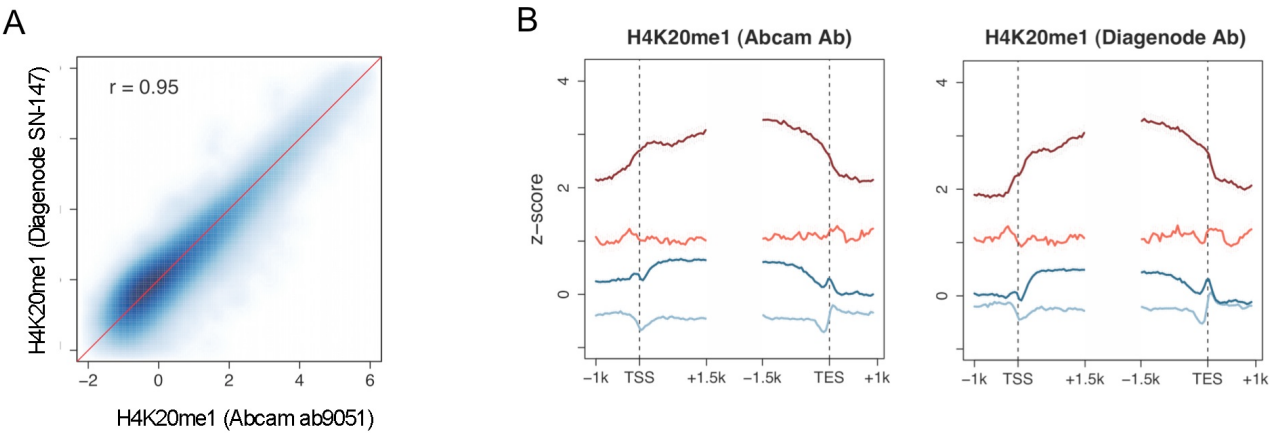

Supplement: Figure S7 — Consistency of different H4K20me1 antibodies in ChIP. (A) Correlation between ChIP-chip signals using antibodies to H4K20me1 from Abcam and Diagenode (r = 0.95). (B) Plots of H4K20me1 signal across the TSS (transcript start site) and TES (transcript end site) of genes on X (red) and autosomes (blue). Genes in the top 20% of expression (dark shades) and bottom 20% (light shades) are plotted separately. Plots generated using data from the two antibodies show similar patterns. (PDF) [file pgen.1002933.s007.pdf]
